# Comprehensive correlation analysis for super-resolution dynamic fingerprinting of cellular compartments using the Zeiss Airyscan detector

**DOI:** 10.1038/s41467-018-07513-2

**Published:** 2018-11-30

**Authors:** L. Scipioni, L. Lanzanó, A. Diaspro, E. Gratton

**Affiliations:** 1 Laboratory for Fluorescence Dynamics, Department of Biomedical Engineering, University of California, Irvine, 92697 CA USA; 2 Nanoscopy, Istituto Italiano di Tecnologia, Genoa, 16163 Italy; 3 Nikon Imaging Center, Istituto Italiano di Tecnologia, Genoa, 16163 Italy

## Abstract

The availability of the Airyscan detector in the Zeiss LSM 880 has made possible the development of a new concept in fluctuation correlation spectroscopy using super-resolution. The Airyscan unit acquires data simultaneously on 32 detectors arranged in a hexagonal array. This detector opens up the possibility to use fluctuation methods based on time correlation at single points or at a number of points simultaneously, as well as methods based on spatial correlation in the area covered by the detector. Given the frame rate of this detector, millions of frames can be acquired in seconds, providing a robust statistical basis for fluctuation data. We apply the comprehensive analysis to the molecular fluctuations of free GFP diffusing in live cells at different subcellular compartments to show that at the nanoscale different cell environments can be distinguished by the comprehensive fluctuation analysis.

## Abstract (web summary)

Different fluorescence fluctuation methods require specialized equipment and cannot be performed simultaneously. Here the authors present an implementation that includes many advanced fluorescence fluctuation techniques in a single analysis, by exploiting the fast and widely used Zeiss Airyscan detector.

## Introduction

Fluorescence fluctuation techniques have been employed in biophysical research for many years^1–7^ and, in the past decade, several new concepts and implementations of fluctuation spectroscopy were introduced in order to answer specific questions regarding complex dynamic behaviors in cellular systems. For instance, techniques such as the pair correlation function^8–11^ (pCF and 2D-pCF) are capable of detecting diffusion barriers and molecular connectivity, while number and brightness analysis^12^ (N&B) can be used for mapping concentration and oligomerization states of the diffusing probe.

Unfortunately, the implementation and spatiotemporal sampling requirements of each of these techniques prevent the simultaneous use of many of these fluorescence fluctuation methods, such as, for instance, image-derived mean square displacement^13,14^ (iMSD) and fluorescence correlation spectroscopy^15,16^ (FCS). Specifically, iMSD exploits spatiotemporal correlations in order to assess the diffusion modality (e.g. free diffusion, confinement or super-diffusion, etc.) and, being based on imaging, has a slow temporal sampling (millisecond/ tens of millisecond) and needs a relatively large region of interest (ROI), therefore averaging events in an area of tens of micrometers squared. On the other hand, FCS is performed by measuring the fluctuations on a single point at the optical resolution limit (typically a few hundreds of nanometers) with a very high temporal sampling (e.g. in the microsecond/nanosecond regime).

FCS has been also used with super-resolution techniques, such as stimulated emission depletion^17–19^ (STED) and separation of photons by lifetime tuning^1,20,21^ (SPLIT), achieving a size of the illumination volume of ~80 nm for EGFP diffusing in living cells. Spot-variation FCS^22–24^ is another FCS-based technique, which consists of a sequentially acquiring FCS dataset while varying the extension of the acquisition volume in order to assess the environment organization. This technique can distinguish between diffusion through a meshwork- or microdomain-like structure. Despite being powerful, spot-variation FCS needs a dedicated microscopy setup capable of changing the illumination volume, and the measurements at different spot sizes are obtained sequentially. Other implementations of spot-variation FCS have been proposed using camera-based systems^25–27^, although the acquisition speed, when compared to single-point detectors, is much lower.

Ultimately, fluorescence fluctuation techniques are generally implemented one at a time, due to the need for a dedicated setup (e.g. spot-variation FCS), a fast temporal sampling (e.g. FCS) or a spatial sampling (e.g. pCF, iMSD).

Comprehensive correlation analysis (CCA), described in this paper, aims at providing an implementation that includes many advanced fluorescence fluctuation techniques in a single, simultaneous analysis by exploiting a fast detector array, namely the Zeiss Airyscan^28^. The Zeiss Airyscan detector operates like a nanocamera with a very high frame rate (about one million frames per second). In the following, we will introduce Airyscan-CCA as the allowing technology for the simultaneous implementation of advanced fluorescence fluctuation techniques in a single, super-resolved ROI. The simultaneous acquisition of fluorescence fluctuations in a multiple detector array measures many biophysical dynamical properties that can be used to create a local dynamic picture of fluorescent or fluorescently labeled probes, and of the environment and obstacles the probes are diffusing through. Among the measures obtained in a single measurement are the oligomerization state (N&B), the diffusion coefficient map and concentration (FCS), the diffusion modality (iMSD), the characterization of barriers to diffusion (2D-pCF) and the nanoscale organization of the environment (spot-variation FCS). This Airyscan nanocamera is a truly unique detector that makes available a spatial and a temporal scale that cannot be reached with any of the current super-resolution microscopy technologies using large-frame cameras. One research field that could strongly benefit from this technique is the study of the dynamics and structure of different cell compartments, including the nucleus of living cells.

## Results

**Fig. 1:**
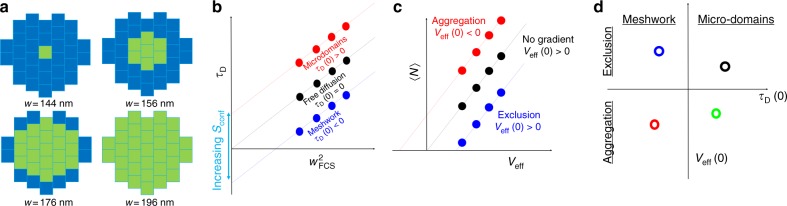
**a** Schematic of the location of the detectors used (green) for obtaining variable waists of the PSF for the spot-variation analysis, together with measured waist relative to each configuration. **b** Schematic of the spot-variation principle for the diffusion time. The linear fitting (dashed lines) of the measured diffusion time as a function of the effective waist squared returns an intercept value. *τ*_D_(0) < 0 in the case of diffusion in a meshwork, *τ*_D_(0) = 0 for free diffusion and *τ*_D_(0) > 0 in the case of diffusion in microdomains. **c** Schematic of the spot-variation principle for the average number; the linear fitting (dashed lines) of the measured number as a function of the effective volume will return an intercept value *V*_eff_(0) 0 in the case of aggregation, *V*_eff_(0) > 0 for exclusion and *V*_eff_(0) = 0 if no gradient is present. **d** Scheme of intercept plot. Each measurement results in a single point in the intercept plot, which shows if the environment is meshwork- (red and blue) or microdomain-like (black and green) and if the diffusing probe is undergoing exclusion (blue and black) or aggregation (red and green)

The Zeiss Airyscan is an array of 32 GaAsP PMT detectors arranged in an hexagonal pattern (shown in Fig. 1) that can reach a sampling speed of up to 1.28 µs per image. A peculiarity of this detector is that the microscope point spread function (PSF) is projected in the center of the array so that each detector acts like a pinhole of approximately 0.2 Airy Units (AU), and is therefore capable of achieving super-resolution as with the image scanning microscopy^29,30^ (ISM) principle. The sampling speed is sufficient to capture the diffusion of free EGFP in solution and in the cell interior, while the spatial extension of the detector (~50 nm pixel size, resulting in an ROI diameter of about 270 nm) allows for the parallel implementation of advanced spatiotemporal correlation techniques such as 2D-pCF and iMSD. The implementation of the Airyscan-CCA is based on measuring spatiotemporal auto- and cross-correlation functions among specific pixels of the Airy detector. In the following we present a series of theoretical and technological challenges that must be overcome in order to successfully implement the CCA technique in the Airy detector. Although other detectors exist that could in principle be used for implementing CCA, for instance SPAD detector arrays^31^, the Airyscan has the advantage of being commercially available and, by exploiting GaAsP PMTs, shows a better quantum efficiency with respect to SPAD.

### Analog detector correction

The substantial issue encountered in the implementation of fluctuation techniques with the Airy detector is the analog nature of this detector. Analog detectors are known for returning incorrect values for the amplitude of the FCS correlation function due to the presence of a detector offset, gain and variance, which contribute in a non-trivial way to the final correlation function^32^. Let us consider a temporal signal, which is a combination of our signal of interest *I*_1_(*t*) and an offset signal *I*_0_(*t*) so that *I*(*t*) = *I*_1_(*t*) + *I*_0_(*t*). The autocorrelation function used to obtain the FCS curve can be written in the following fashion: $G\left( \tau \right) = \frac{{\left\langle {\delta I(t)\delta I(t + \tau )} \right\rangle }}{{\left\langle {I(t)} \right\rangle ^2}}$ where the brackets denote the temporal average of the signal and *δI*(*t*) = *I*(*t*)−〈*I*(*t*)〉 = *δI*_1_(*t*) + *δI*_0_(*t*). Substituting the sum of the signals we obtain $G\left( \tau \right) = \frac{{\left\langle {\delta I_1(t)\delta I_1(t + \tau )} \right\rangle + \left\langle {\delta I_0(t)\delta I_1(t + \tau )} \right\rangle + \left\langle {\delta I_1(t)\delta I_0(t + \tau )} \right\rangle + \left\langle {\delta I_0(t)\delta I_0(t + \tau )} \right\rangle }}{{\left\langle {I_1\left( t \right) + I_0(t)} \right\rangle ^2}}$ and assuming no cross-correlation between the offset signal and the signal arising from the fluorescent probes we cancel out the cross terms, obtaining $G\left( \tau \right) = \frac{{\left\langle {\delta I_1(t)\delta I_1(t + \tau )} \right\rangle + \left\langle {\delta I_0(t)\delta I_0(t + \tau )} \right\rangle }}{{\left( {\left\langle {I_1\left( t \right)} \right\rangle + \left\langle {I_0\left( t \right)} \right\rangle } \right)^2}},$ where the 〈*δI*_0_(*t*)*δI*_0_(*t* + *τ*)〉 and 〈*I*_0_(*t*)〉 terms are solely related to the offset signal, which can be characterized by acquiring and analyzing a dark dataset with no fluorescence fluctuation signal, for instance by switching off the excitation laser. Once characterized and stored, these terms can be included in the calculation in order to obtain the corrected form for *G*_1_(*τ*)

$$G_1(\tau ) 	= \frac{{\left\langle {\delta I(t)\delta I(t + \tau )} \right\rangle - \left\langle {\delta I_0(t)\delta I_0(t + \tau )} \right\rangle }}{{\left( {\left\langle {I(t)} \right\rangle - \left\langle {I_0\left( t \right)} \right\rangle } \right)^2}} \\ 	 = \frac{{\left\langle {\delta I_1(t)\delta I_1(t + \tau )} \right\rangle }}{{\left( {\left\langle {I_1\left( t \right)} \right\rangle } \right)^2}}.$$

With this correction, which is straightforward to extend to the cross-correlation function between distinct detectors and groups of detectors, we were able to account for the detector dark noise offset and variance and the cross-talk between detectors. Note that the gain, being merely a multiplication factor, is canceled out in the autocorrelation function:

$$G_{A \cdot I(t)}\left( \tau \right) 	= \frac{{\left\langle {A \cdot \delta I(t) \cdot A \cdot \delta I(t + \tau )} \right\rangle }}{{\left\langle {A \cdot I(t)} \right\rangle ^2}} = \frac{{A^2 \cdot \left\langle {\delta I(t) \cdot \delta I(t + \tau )} \right\rangle }}{{A^2 \cdot \left\langle {I(t)} \right\rangle ^2}} \\ 	 = \frac{{\left\langle {\delta I(t) \cdot \delta I(t + \tau )} \right\rangle }}{{\left\langle {I(t)} \right\rangle ^2}} = G_{I(t)}\left( \tau \right).$$

We are now capable of determining the apparent molecular brightness of the diffusing probes with an analog detector, which can be obtained from $B_{\mathrm{analog}} = \frac{{\left\langle {I_1(t)} \right\rangle \cdot G_1(0)}}{{t_{\mathrm{sample}}}}$ where *t*_sample_ is the sampling time, as well as the average number of molecules ⟨*N*⟩ diffusing in the illumination volume $\left\langle N \right\rangle = \frac{\gamma }{{G_1(0)}},$ where *γ* is a shape factor dependent on the functional form of the illumination volume. Note that the brightness, as opposed to the *G*_1_(0) value, is influenced by the gain of the detector in the average intensity ⟨*I*_1_(*t*)⟩ term. Assuming a simple proportionality between the number of photons emitted by the probe, *N*_ph_, and the average intensity, the gain *S*_detector_ can be written as $S_{\mathrm{detector}} = \frac{{\left\langle {I_1(t)} \right\rangle }}{{N_{\mathrm{ph}}}}$ and, consequently $B_{\mathrm{analog}} = \frac{{N_{\mathrm{ph}} \cdot S_{\mathrm{detector}} \cdot G_1\left( 0 \right)}}{{t_{\mathrm{sample}}}} = B_{\mathrm{app}} \cdot S_{\mathrm{detector}}$ where $B_{\mathrm{app}} = B_{\mathrm{real}} \cdot {\mathrm{QE}}$, where QE is the combined quantum yield of the probe and the optical system with a certain set of acquisition parameters and *B*_real_ is the brightness of the probe. *S*_detector_ can be obtained by a calibration with a sample with known brightness, for instance a sample yielding Poisson noise, in order to obtain *B*_app_^32^. Brightness analysis can be used to obtain information about the oligomerization states of the diffusing probe, while the number can be used to retrieve the concentration of diffusing molecules in the illumination volume. It is worth noting that *B*_analog_ can be directly used to obtain the oligomerization state of the probe, provided that the brightness of the monomeric state of the probe is obtained under the same experimental conditions.

### Temporal correlation techniques

The most straightforward techniques that can be implemented in the Airyscan-CCA are temporal correlation techniques such as FCS and spot-variation FCS^25–27^, which rely merely on the autocorrelation function of the temporal fluorescence signal.

Spot-variation FCS is commonly implemented by sequentially acquiring FCS curves with different illumination volumes and plotting the fitted value of the diffusion coefficient as a function of the waist squared. In the Airy detector, we consider four volumes obtained by summing the signals coming from selected detectors, as shown in Fig. 1a. This operation is equivalent to opening and closing a virtual pinhole and, since every detector is 0.2 Airy units, we have access to super-resolution PSFs with waist ranging from 144 to 196 nm. For each of the volumes, we obtain a value of the diffusion time *τ*_D_ that we plot as a function of the waist squared (Fig. 1b). Then, we fit the experimental points with a linear function and we consider its intercept at *w*^2^ = 0. As discussed in ref. ^22^, if the intercept is positive, the probes are diffusing in a microdomain-like environment, while if the intercept is negative the environment structure is meshwork-like. In both cases, the strength of confinement *S*_conf_ increases when the intercept absolute value increases^22^.

Thanks to the analog correction we discussed, we can also consider the amplitude of the correlation function, which depends on the number of molecules as a function of the effective volume of acquisition $V_{\mathrm{eff}} = \pi ^{3/2}w^3A$, a similar concept has been exploited elsewhere^1^. Here, *A* is the ratio between the waist in the axial and longitudinal direction, which can be measured by 3D imaging of a sample of fixed 20 nm fluorescent beads and is calibrated for every volume used for the spot-variation analysis. The 3D Gaussian fitting of the beads signal returns a value of *A* = 2.97 for the larger volume in the setup used. The *N*(*V*_eff_) curve depends on the change in the number of diffusing molecules when varying the acquisition volume. Therefore, if any part of the volume is inaccessible to diffusing molecules, the curve will intercept the *x*-axis at a positive value that will increase when the volume excluded to the diffusing molecules increases. On the other hand, if the diffusing molecules are more concentrated in the center of the acquisition volume, the intercept will be negative, increasing its absolute value as the concentration gradient increases.

In summary, our enhanced spot-variation FCS analysis returns two values, the intercept of the *τ*_D_(*w*^2^) for *w*^2^ = 0, which gives information about the environmental organization, and the intercept of the *N*(*V*_eff_) for *N* = 0, which gives information about the exclusion (or aggregation) of diffusing molecules in the acquisition volume. From these two parameters, we identify a point in a 2D plot that we named the intercept plot, in which the *x*-axis is the *V*_eff_(0) value and the *y*-axis is the *τ*_D_(0) values. The position of the point in the intercept plot will reveal the organization of the environment sensed by the diffusing probes.

So far, we have only utilized the temporal autocorrelation function calculated for one or for the sum of multiple detectors (Fig. 1a) and the representation in the Intercept plot (Fig. 1d) after linear extrapolation of the diffusion time as a function of the effective waist squared (Fig. 1b) and of the average number <*N*> as a function of the effective volume (Fig. 1c).

Spatiotemporal correlation techniques can be implemented thanks to the spatial extension of the detector. In the following we discuss the implementation of 2D-pCF and iMSD within the CCA technique.

### 2D-pCF

In 2D-pCF analysis, the algorithm calculates the cross-correlation function between the time signal acquired at one detector (the central point) and the time signal acquired at detectors located at a certain distance from the central one. The 2D-pCF function gives information on the existence of barriers or preferential pathways to diffusion by analyzing the correlation between spatially separated locations. To calculate the 2D-pCF we use the following correlation function

$$G\left( {\tau ,{\boldsymbol{r}}} \right) = \frac{{\left\langle {I(t,0)I(t + \tau ,{\boldsymbol{r}})} \right\rangle }}{{\left\langle {I(t,0)} \right\rangle \cdot \left\langle {I(t,{\boldsymbol{r}})} \right\rangle }} - 1$$

**Fig. 2:**
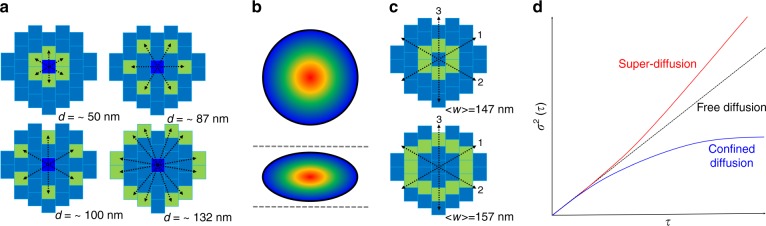
**a** Schematic of the pixels used for computing the 2D pair correlation function. The cross-correlation is computed between the central pixel (blue) and the highlighted pixels (green), the 2D-pCF can be computed at four different distances, stated next to the relative configuration. **b** Schematic example of 2D-pCF in the case of diffusion without barriers (top) and with barriers (bottom) affecting the diffusion of the probe. **c** Schematic showing the location of the detectors used (green) for the iMSD analysis in ring 1 (top) and ring 2 (bottom). The measured average waist relative to each configuration is shown in the figure. Dashed black arrows refer to the direction along which the spatiotemporal correlation is computed. **d** Schematic of the iMSD principle. The fitted variance of the spatiotemporal correlation function increases linearly in the case of free diffusion (dashed black line) and bends upwards or downwards in the case of super-diffusion (solid red curve) or confined diffusion (solid blue line), respectively

In our implementation, the central detector occupies the 0 position in the hexagonal array of the Airy detector and we can define four radii corresponding to four groups of detectors as shown in Fig. 2a. The cross-correlation between the central detector and a detector at a certain angle *θ*and distance *r* will return the *G*_*θ*_(*τ*, *r*) function. The functions computed at all possible angles at a fixed distance is represented in a log correlation time scale and is used for representing the local diffusion anisotropy and the direction of the barrier for diffusion if one exits, as explained elsewhere^8,9^ (Fig. 2b). Note that, since the waist of the PSF of the single detector increases with their distance from the optical axis (Supplementary Notes), this approach is the simplest way to implement 2D-pCF, since any other configuration would result in having detectors with the different waist at the same distance, introducing a bias in the analysis. In the Supplementary Notes, we show how it is possible to account for this effect and construct a connectivity map of diffusion inside the ROI of the Airyscan detector.

### iMSD

As mentioned before, every single detector in the Airy detectors displays a different value of waist according to its distance from the optical axis, therefore the implementation of iMSD should consider only groups of detectors with comparable waists. Failure to do so will result in a bias of the iMSD curve which will appear as an artificially confined motion. Such detectors are highlighted in Fig. 2c. and will be referred to as Ring 1 and Ring 2. For the analysis, only Ring 2 is considered since the detectors occupy a larger area, providing a better sampling of the spatiotemporal correlation function. In order to account for the presence of anisotropy, we consider a linear iMSD in three different directions, as shown in Fig. 2c. iMSD consists in fitting the broadening of the spatiotemporal correlation function *G*(*ξ*, *η*, *τ*) to a 2D Gaussian function and plotting the value of the variance *σ*^2^ as a function of the temporal lag *τ*. Since the spatiotemporal correlation function is sampled in only nine spatial lags for Ring 2, the iMSD curve considered in this work is retrieved from the analysis of the *G*(0, 0, *τ*), namely the change in amplitude of the Gaussian as a function of the temporal lag, the fitting of which yields more robust results.

As determined elsewhere^14^, we can write, for 2D diffusion, $G\left( {0,0,\tau } \right) = \frac{\gamma }{{N\pi \sigma ^2(\tau )}}$ therefore, the iMSD curve *σ*^2^(*τ*) can be written as $\sigma ^2\left( \tau \right) = \frac{C}{{N \cdot G\left( {0,0,\tau } \right)}}$where *C* is a constant scale factor which we obtain from the calibration. In our implementation, the *G*(*ξ*, *η*, *τ*) is computed along three directions, therefore we consider only one spatial dimension and the function can be written as *G*_*θ*_(*χ*, *τ*) where *θ* is the angle which defines the direction considered and *χ* is the spatial lag along that direction. *N* denotes the average number of molecules, which we computed as $N = \frac{1}{{\left\langle {G\left( 0 \right)} \right\rangle _{\mathrm {Ring}}}}$, where 〈*G*(0)〉_Ring_ is the average of the *G*(0) values of the detectors of the ring that we consider. Finally, the iMSD function computed at the angle *θ*, denoted as $\sigma _\theta ^2\left( \tau \right)$, is calculated as

$$\sigma _\theta ^2\left( \tau \right) = C\frac{{\left\langle {G\left( 0 \right)} \right\rangle _{\mathrm {Ring}}}}{{G_\theta \left( {0,\tau } \right)}}$$

### Examples of application of the CCA analysis

**Fig. 3:**
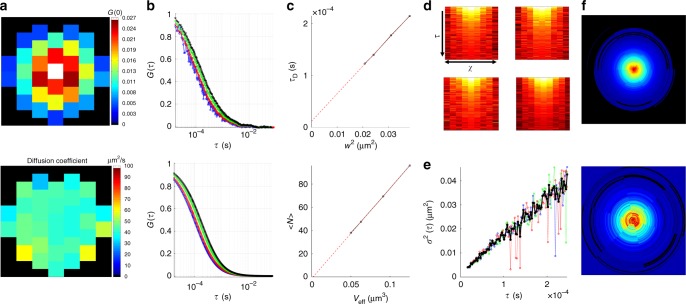
**a** Map of the fitted *G*(0) for the single detectors (top) together with intensity distribution (bottom). **b** FCS curves computed from a typical measure in the cytoplasm (top) together with the fitting with a single component diffusion model (bottom), the colors refer to rings the volumes used for the spot-variation FCS (volume increasing going from blue, red, green and black, respectively, as shown in Fig. 1a), the curves are normalized to the fitted G(0) values, **c** spot-variation curves obtained from the fitting of b, fitted diffusion time as a function of the waist squared (top) and average number as a function of the effective volume (bottom). **d** ${{G}}({{\chi }},{{\tau }})$ carpets for directions 1 (top left), 2 (top right) and 3 (bottom left) together with the average of all three directions (bottom right). **e** iMSD curve relative to directions 1–3 (blue, red, and green curves, respectively) and average (black curve). **f** 2D-pCF function for distance 1 (top) and 4 (bottom)

The CCA analysis has been applied to several intracellular compartments in NIH-3T3 cells stably expressing EGFP. The compartments we are interested in characterizing are the unstructured and structured cytoplasm, the center of the nucleolus and heterochromatin- and euchromatin-rich regions in the nucleoplasm. These regions were determined by imaging an EGFP-expressing stable cell line labeled with a DNA stain, the criteria of selection for the regions used for this analysis are described in the Supplementary Notes and an example of the CCA analysis for unstructured cytoplasm is shown in Fig. 3. The spot-variation analysis (Fig. 3b, c) shows a positive intercept for the diffusion time, therefore placing the unstructured cytoplasm in the microdomain structured regime according to the classification of the intercept plot, and a positive intercept for the effective volume, which means the presence of an excluded volume. For this case, the iMSD analysis (Fig. 3d, e) shows that the diffusion is free and isotropic along the three directions, as confirmed also by the isotropic shape of the pCF curves (Fig. 3f).

**Fig. 4:**
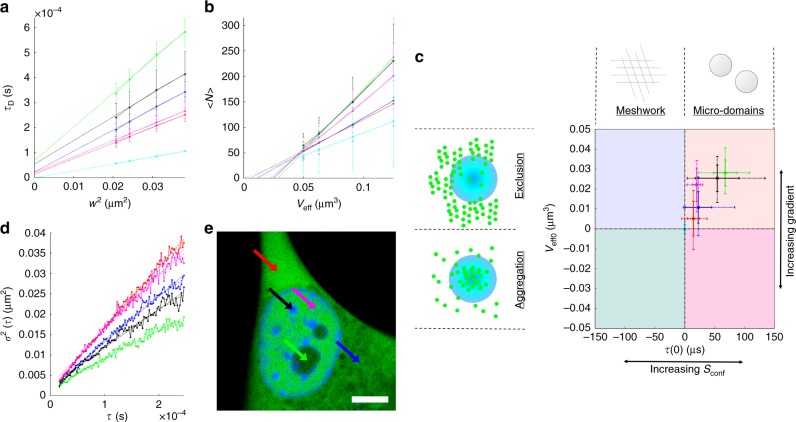
Spot-variation curves for diffusion time (**a**) and number (**b**) from which the intercept plot (**c**) is constructed, along with the iMSD curves (**d**) for several cellular compartment shown in **e**, namely unstructured (red) and structured (blue) cytoplasm, heterochromatin- (black) and euchromatin-rich (magenta) nucleoplasm, nucleolus (green) and free EGFP in solution (cyan). Dashed lines in **a**, **b** show the linear fitting of the curves while dotted and dashed lines in **d** show the fitting with a free diffusion and a confined model, respectively. Scale bar in **e** is 5 μm. Solid error bars denote the standard deviation computed from 25 cells, while dashed error bars denote the extremes of the distribution

The analyses of all measured compartments were compared and the overall result is shown in Fig. 4. The spot-variation analysis curves (Fig. 4a, b) were used for constructing the intercept plot (Fig. 4c), while the fitting for the iMSD returned a confined mode of diffusion for all compartments, with the exception of the unstructured cytoplasm which returned a free mode of diffusion with a diffusion coefficient of 37 μm^2 ^s^−1^. As expected, the control sample of EGFP in solution is located in the center of the plot (Fig. 4c, cyan), corresponding to an unstructured, unexcluded environment, while all the other compartments measured occupy the microdomain/exclusion quadrant. Cytoplasm compartments (Fig. 4c, red and blue) show a smaller confinement and a smaller excluded volume compared to nuclear compartments (Fig. 4c, magenta, black and green), which also show an increase in confinement strength going from euchromatin-rich to heterochromatin-rich regions, ultimately leading to the nucleolus, which, being the most compact compartment, displays the highest degree of exclusion. Interestingly, heterochromatin-rich regions (Fig. 4c, black) display a high variability in the strength of confinement, which can be linked to the degree of compaction of the region, therefore making CCA an invaluable tool for the super-resolution characterization of chromatin compaction in living cells.

Regarding iMSD, the parameters obtained from the fitting of the curve are summarized in Table 1 and show that the confinement length is progressively increasing from euchromatin, structured cytoplasm, heterochromatin or the nucleolus, in keeping with what is shown in Fig. 4c. All the compartments considered in the analysis fit in the top right quadrant of the intercept plot (Fig. 4c), which corresponds to the exclusion and microdomain organization. These results are anticipated since EGFP is a small protein (27 kDA, ~3 nm), therefore is not sensitive to a meshwork-like environment. EGFP is also an inert probe, not interacting with other cellular structures, therefore not showing binding behavior which would appear in the aggregation quadrants.

**Table 1:** Table of values obtained from the iMSD fitting for free diffusion (*D*_free_) and confined diffusion (*L*_conf_) for each compartment, along with the intercept value in *τ* = 0 (*σ*^2^(0))

	*D*_free_ (μm^2 ^s^−1^)	*σ*^2^(0) (μm^2^)	*L*_conf_ (nm)
Cytoplasm (unstructured)	36.9	0.00048	/
Cytoplasm (structured)	27.7	0.00036	417
Nucleus (euchromatin)	33.2	0.00050	491
Nucleus (heterochromatin)	23.2	0.00057	370
Nucleus (nucleolus)	17.2	0.00052	297

## Discussion

The study of protein dynamics and the use of EGFP as a probe for investigating the structure of cellular compartments have been used for decades^1,2,4,6,11,13,33,34^ while, on a parallel effort, many microscopy techniques have been developed from the super-resolution imaging field (STED, ISM…) and from the fluorescence fluctuations field (pCF, iMSD, spot-variation FCS, N&B…) in order to provide increased spatiotemporal resolution to address dynamic as well as structural information of the cellular environment. Although many studies have focused on the coupling of STED with fluorescence fluctuation techniques, our work is, at the best of our knowledge, the first coupling ISM concepts to such techniques enabling, on a different spatial scale, the super-resolution investigation of many dynamic properties in a single analysis in a few seconds. CCA analysis displays low photobleaching, low phototoxicity and high reproducibility with the use of low laser power and, most importantly, this technique does not require either specific expertise or a custom-made setup and is readily available to be implemented on any microscope equipped with an Airyscan detector. Applying CCA on a fluorescent or fluorescently-labeled sample can provide a simultaneous quantification of a number of biophysical parameters related to the probe and to the diffusing environment, such as oligomerization state, concentration, diffusion coefficient, diffusion modality, diffusion anisotropy, environment organization and exclusion/aggregation information, all with a resolution down to ~1.4 times smaller than the diffraction limit.

We demonstrated that CCA is capable of exhaustively characterizing the diffusion of EGFP in many cellular compartments, providing a fingerprinting tool for the investigation of structural and dynamic properties of the subcellular environment.

## Methods

### Microscope

All measurements were performed on a Zeiss LSM 880 microscope equipped with the AiryScan detector, an Argon laser (Melles-Griot) for 488 nm excitation and a Zeiss Plan-Apochromat 63×/1.4 NA DIC M27 Oil objective. The acquisition modality was set to Spot acquisition with 2.46 µs per time point, bit depth was set at 16 bits, gain at 750 and the pinhole aperture was set to max. All files were successively saved in the ome.tiff format to be processed from our MATLAB routine. The microscope is equipped with temperature and CO_2_ controls, that were kept at 37 °C and 5%, respectively.

### Cells

A stable EGFP expressing cell line of NIH-3T3 cells was used for all the experiments^35^. Briefly, a NIH-3T3 cell line was transfected with the pEF5/FRT/EGFP plasmid and stabilized using the Flip-In method following manufacturer’s recommendations (Life Technologies). Cells were plated in Ibidi µ-slide 8-well plates with 1.5H glass bottom, allowed to grow overnight and stained with 5 µl NucBlue (Thermofisher) diluted in 250 µl of HBSS per well, left in the incubator for 10 min, rinsed 3 times and left recovering 30 min in cell culture medium in the incubator. The cells were used for no more than two days after being plated.

### Calibration

First, the laser beam is aligned to the center of the Airyscan detector by imaging a fluorescent plastic slide (Chroma). Once focused, the beam is either manually or automatically centered. Successively, a sample of EGFP (BioVision) at a concentration varying from 0.1 to 1 µM is acquired in the same 8-well plates used for the experiments with cells with a laser power of 5% (~50 µW) for 40 million time points in three successive acquisitions. The 8-well plates were previously coated with BSA (2 mg ml^−1^ for 2 h, rinsed and let to dry) in order to prevent EGFP from sticking to the glass. Before and after each acquisition, an FCS curve is obtained by means of a SPC detector (average of ten 2 s long acquisitions with a laser power of ~40 µW) and the *G*(0) value is recorded for the number calibration. Finally, a dark dataset is acquired with the laser switched off for 10 million time points. Calibration files could be acquired also at the end of the acquisition session in order to evaluate system stability.

### Acquisition

The cells were randomly chosen; a two-color image was taken in order to localize the experimental point of acquisition; then a single-point acquisition of 10 million time points (~25 s) with a laser power of 1.5% (~18 µW) was performed in the area and finally a second two-colors image was taken in order to evaluate if the cell underwent any movement or conformational change. Cells undergoing movement were discarded, no conformational change was noticed after acquiring with these settings.

### Analysis

All the data calculations and figures for this paper were done with a custom-written MATLAB code. The same routines are also available as a self-standing Airyprogram.exe file for Windows 7 or higher running on a PC. This program can directly use the czi file produced by the ZEN software in the Zeiss LSM 880 Airyscan. The Airyprogram.exe code is available at https://www.lfd.uci.edu/globals/ under Globals for Airyscan. A tutorial for using this software is also available at the same site in PDF form.

## Electronic supplementary material

Supplemental Information

Reporting Summary

## Data Availability

The datasets generated during and/or analyzed during the current study are available from the corresponding author on reasonable request.

## References

[1] Lanzanò, L. et al. Measurement of nanoscale three-dimensional diffusion in the interior of living cells by STED-FCS. *Nat. Commun*. **8**, 65 (2017). 10.1038/s41467-017-00117-2 PMC5500520 28684735

[2] Dross N (2009). Mapping eGFP oligomer mobility in living cell nuclei. PLoS One.

[3] Brown CM (2008). Raster image correlation spectroscopy (RICS) for measuring fast protein dynamics and concentrations with a commercial laser scanning confocal microscope. J. Microsc..

[4] Scipioni L, Di Bona M, Vicidomini G, Diaspro A, Lanzanò L (2018). Local raster image correlation spectroscopy generates high-resolution intracellular diffusion maps. Commun. Biol.

[5] Digiacomo L, Digman MA, Gratton E, Caracciolo G (2016). Development of an image mean square displacement (iMSD)-based method as a novel approach to study the intracellular trafficking of nanoparticles. Acta Biomater..

[6] Pack C, Saito K, Tamura M, Kinjo M (2006). Microenvironment and effect of energy depletion in the nucleus analyzed by mobility of multiple oligomeric EGFPs. Biophys. J..

[7] Scipioni, L., Gratton, E., Diaspro, A. & Lanzan, L. Phasor analysis of local ICS detects heterogeneity in size and number of intracellular vesicles. *Biophys. J.* **111**, 619–629 (2016). 10.1016/j.bpj.2016.06.029 PMC4982927 27508445

[8] Malacrida, L., Rao, E. & Gratton, E. Comparison between iMSD and 2D-pCF analysis for molecular motion studies on in vivo cells: the case of the epidermal growth factor receptor. *Methods*. 10.1016/j.ymeth.2018.01.010 (2018). 10.1016/j.ymeth.2018.01.010 PMC5999564 29501424

[9] Di Rienzo C, Cardarelli F, Di Luca M, Beltram F, Gratton E (2016). Diffusion tensor analysis by two-dimensional pair correlation of fluorescence fluctuations in cells. Biophys. J..

[10] Cardarelli EH, Measuring F (2011). The flow of molecules in cells. Biophys. Rev..

[11] Hinde E, Cardarelli F, Digman MA, Gratton E (2010). In vivo pair correlation analysis of EGFP intranuclear diffusion reveals DNA-dependent molecular flow. Proc. Natl. Acad. Sci. USA.

[12] Digman MA, Dalal R, Horwitz AF, Gratton E (2008). Mapping the number of molecules and brightness in the laser scanning microscope. Biophys. J..

[13] Di Rienzo C, Piazza V, Gratton E, Beltram F, Cardarelli F (2014). Probing short-range protein Brownian motion in the cytoplasm of living cells. Nat. Commun..

[14] Di Rienzo C, Gratton E, Beltram F, Cardarelli F (2013). Fast spatiotemporal correlation spectroscopy to determine protein lateral diffusion laws in live cell membranes. Proc. Natl. Acad. Sci. USA.

[15] Saffarian S, Elson EL (2003). Statistical analysis of fluorescence correlation spectroscopy: the standard deviation and bias. Biophys. J..

[16] Elson EL (2011). Fluorescence correlation spectroscopy: past, present, future. Biophys. J..

[17] Hell, S. W. & Wichmann, J. Stimulated-emission-depletion fluorescence microscopy. *Opt. Lett*. 10.1364/OL.19.000780 (1994). 10.1364/ol.19.000780 19844443

[18] Vicidomini, G. et al. STED nanoscopy with time-gated detection: theoretical and experimental aspects. *PLoS ONE*. 10.1371/journal.pone.0054421 (2013). 10.1371/journal.pone.0054421 PMC3548795 23349884

[19] Harke, B. et al. Resolution scaling in STED microscopy. *Opt. Express*. 10.1364/OE.16.004154 (2008). 10.1364/oe.16.004154 18542512

[20] Lanzano, L., Vicidomini, G., Scipioni, L., Castello, M. & Diaspro, A. STED microsc.: explor. fluoresc. lifetime gradients super.-resolut. reduc. illum. intensities. multiphoton microsc. fluoresc. lifetime imaging. *Appl. Biol. Med*. 10.1515/9783110429985-007 (2018).

[21] Lanzanò L (2015). Encoding and decoding spatio-temporal information for super-resolution microscopy. Nat. Commun..

[22] Wawrezinieck L, Rigneault H, Marguet D, Lenne PF (2005). Fluorescence correlation spectroscopy diffusion laws to probe the submicron cell membrane organization. Biophys. J..

[23] Lenne PF (2006). Dynamic molecular confinement in the plasma membrane by microdomains and the cytoskeleton meshwork. EMBO J..

[24] Wenger J (2007). Diffusion analysis within single nanometric apertures reveals the ultrafine cell membrane organization. Biophys. J..

[25] Krieger JW (2015). Imaging fluorescence (cross-) correlation spectroscopy in live cells and organisms. Nat. Protoc..

[26] Liu, H., Dong, C. & Ren, J. Tempo-spatially resolved scattering correlation spectroscopy under dark-field illumination and its application to investigate dynamic behaviors of gold nanoparticles in live cells. *J. Am. Chem. Soc*. 10.1021/ja410284j (2014). 10.1021/ja410284j 24460214

[27] Capoulade, J., Wachsmuth, M., Hufnagel, L. & Knop, M. Quantitative fluorescence imaging of protein diffusion and interaction in living cells. *Nat. Biotechnol*. 10.1038/nbt.1928 (2011). 10.1038/nbt.1928 21822256

[28] Huff, J. The Airyscan detector from ZEISS: confocal imaging with improved signal-to-noise ratio and super-resolution. *Nat. Methods* **12**, i–ii (2015).

[29] Müller CB, Enderlein J (2010). Image scanning microscopy. Phys. Rev. Lett..

[30] Sheppard CJR, Mehta SB, Heintzmann R (2013). Superresolution by image scanning microscopy using pixel reassignment. Opt. Lett..

[31] Sheppard, C. J. R. et al. Image scanning microscopy (ISM) with a single photon avalanche diode (SPAD) array detector. *Opt. Photonics Digit. Technol. Imaging Appl. V*. 10.1117/12.2309825 (2018).

[32] Dalal RB, Digman MA, Horwitz AF, Vetri V, Gratton E (2008). Determination of particle number and brightness using a laser scanning confocal microscope operating in the analog mode. Microsc. Res. Tech..

[33] Gröner N, Capoulade J, Cremer C, Wachsmuth M (2010). Measuring and imaging diffusion with multiple scan speed image correlation spectroscopy. Opt. Express.

[34] Park H, Han SS, Sako Y, Pack CG (2015). Dynamic and unique nucleolar microenvironment revealed by fluorescence correlation spectroscopy. FASEB J..

[35] Torrado, B., Grana, M., Badano, J. L. & Irigoin, F. Ciliary entry of the Hedgehog transcriptional activator Gli2 is mediated by the nuclear import machinery but differs from nuclear transport in being Imp-α/β1-independent. *PLoS ONE*. 10.1371/journal.pone.0162033 (2016). 10.1371/journal.pone.0162033 PMC5007031 27579771

