# Supplementary material for: Comprehensive correlation analysis for super-resolution dynamic fingerprinting of cellular compartments using the Zeiss Airyscan detector
Source: Nat Commun. 2018 Nov 30;9:5120. doi: 10.1038/s41467-018-07513-2 (PMC6269422; doi:10.1038/s41467-018-07513-2)
Supplement: Supplementary file 1 — Supplemental Information [file 41467_2018_7513_MOESM1_ESM.pdf]

SUPPLEMENTARY INFORMATION

Scipioni et al.

SUPPLEMENTARY NOTE 1

Analog correction

After implementing the correction for the analog contribution, we are capable to retrieve the correct values of $G(0)$ for the four volumes used for spot variation FCS, as shown in Supplementary Figure 1. The trend of the $G(0)$ values measured at different EGFP concentration follows the same trend as the $G(0)$ computed from the FCS curves obtained from the SPC detector. The correction provides correct $G(0)$ values over one order of magnitude. The concentration values were chosen in order to comprehend the range of concentration typically found in our samples (0.01/0.03). It's worth noting that employing a NIH-3T3 stable line allows for reproducible concentration values between cells, since the expression of the plasmid is better controlled.

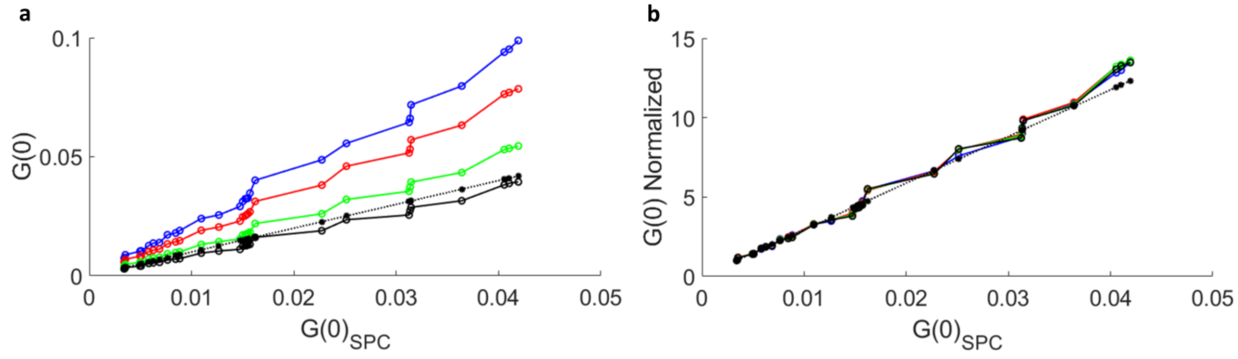

Supplementary Figure 1 (a) Plot of the corrected $G(0)$ values for each waist (blue, red, green and black solid lines, going from smaller to larger waist) as a function of the values of the $G(0)$ measured with the SPC detector (black asterisks, dotted black line) and (b) the same curves in a normalized to the smaller $G(0)$ value for each curve.

Calibration

Supplementary Figure 2 shows an example of calibration, in which the waist of the single detectors are obtained (Supplementary Figure 2 a) together with all the information needed in order to construct the spot-variation FCS (Supplementary Figure 2 b, c) and iMSD (Supplementary Figure 2 d, e) curves, together with a test for pCF symmetry (Supplementary Figure 2 f).

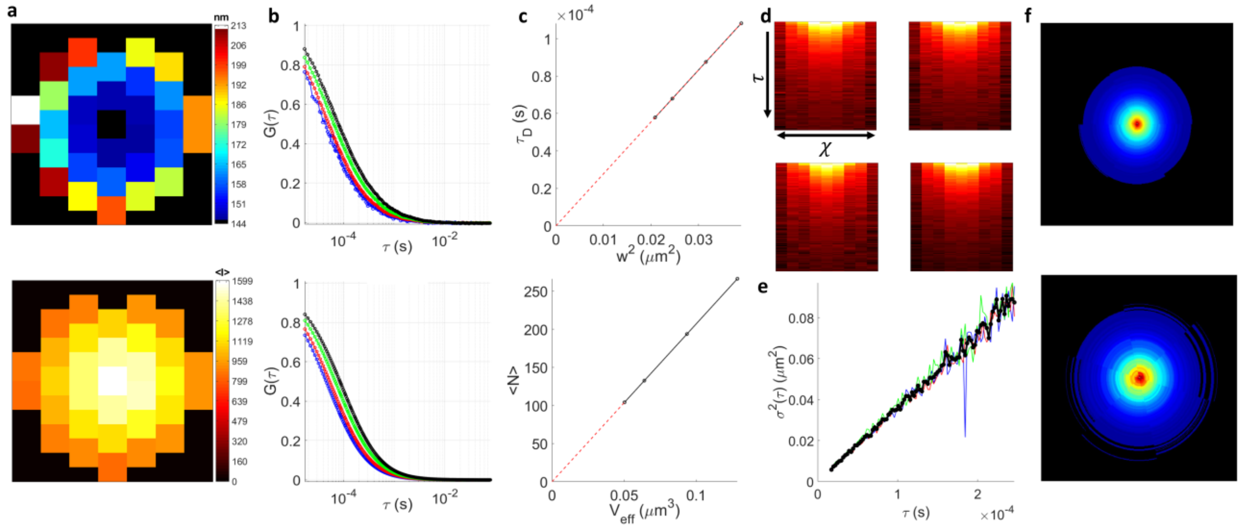

Supplementary Figure 2 a: Map of the fitted waist for each detector (top) together with intensity distribution (bottom). b: Normalized FCS curves computed from a calibration sample of EGFP in solution (top) together with fitting with a single component model (bottom), the colors refer to rings 1 to 4 (blue, red, green and black, respectively, shown in ***Error! Reference source not found.*** *a). c: spot-variation curves obtained from the fitting of b, fitted diffusion time as a function of the waist squared (top) and average number as a function of the effective volume (bottom). d:* $G(\chi,\tau)$ *carpets for directions 1 (top left), 2 (top right) and 3 (bottom left) together with the average of all three directions (bottom right). e: iMSD curve for the directions 1-3 (blue, red and green curves, respectively) and average (black curve). f: 2D-pCF function for distance 1 (top) and 4 (bottom).*

Batch analysis

We compared the results of our measurements to characterize the dynamic fingerprint of each compartment. In Supplementary Figure 3 we show an example of batch analysis obtained from 25 samples of euchromatin-rich nucleoplasm, in which we can see that the iMSD curves are consistent for all these experiments (Supplementary Figure 3 a), as confirmed also by the spot-variation curves for diffusion time (Supplementary Figure 3 b, top), while the amount of excluded volume appears more heterogeneous (Supplementary Figure 3 b, bottom). To better visualize the meaning of the spot-variation fingerprinting, we constructed the intercept plot (Supplementary Figure 3 c), in which we plot the intercept of the excluded volume as a function of the intercept of the diffusion time, therefore describing every experiment with a single point in this plot. The intercept plot consists of four distinct quadrants, each of which describes a particular structural configuration, namely meshwork-like environment (left quadrants), micro-domains environment (right quadrants), exclusion (top quadrants) and aggregation (bottom quadrants). CCA calculated for each compartment will ultimately return the diffusion modality in the compartment (free, confined, super-diffusion etc.) from the iMSD analysis, together with a structural analysis, shown in the intercept plot (Supplementary Figure 3 c).

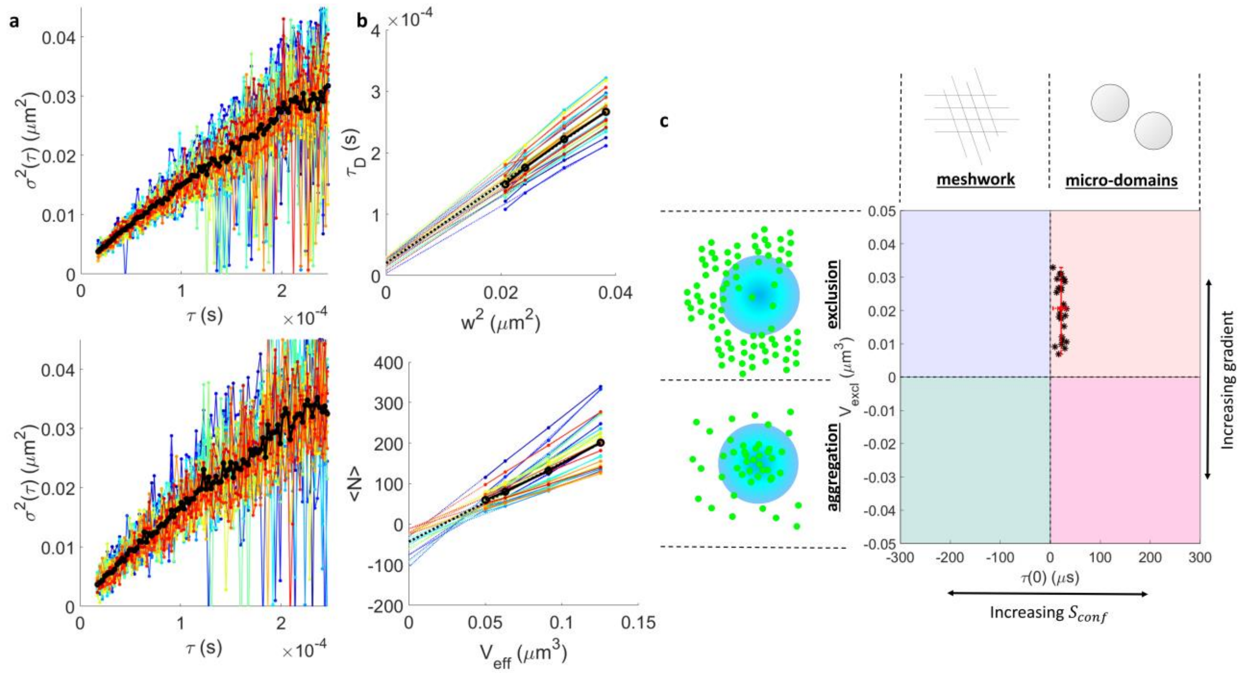

Supplementary Figure 3 a: iMSD curves for Rings 1 (top) and 2 (bottom) obtained from N=25 measurements of euchromatin-rich regions in the nucleus (colored curves) together with average curves (black). b: Spot-variation curves for diffusion time (top) and number (bottom), colored curves refer to single experiments while the black curves refer to the average. c: Intercept plot obtained from the values fitted from b, the black asterisks refer to single experiment while the red dot refer to the average, dotted error bars represent the minimum and maximum values of all experiment while the solid error bars represent the standard deviation. The schemes surrounding the plot show the interpretation of the individual quadrants. Solid error bars denote the standard deviation computed from 25 cells, while dashed error bars denote the extremes of the distribution.

Connectivity mapping

In principle, connectivity maps could not be applied in the traditional sense since the waist of every single detector is different according to its distance from the optical axis. This difference results in an artificially anisotropic map due to the fact that the probe signal lasts for a longer time when the probes travel between larger waists. However, we could overcome this limitation by building a connectivity map of diffusion coefficient instead of using diffusion times. As a theoretical framework we used the dual focus FCS equation [1,2] which, in our implementation, can be written as

$$G_r(\tau) = G_r(0)\frac{1}{\left(1+\frac{4D\tau}{w_{eff}^2}\right)\sqrt{\left(1+\frac{4D\tau}{A^2\cdot w_{eff}^2}\right)}}e^{-\frac{r^2}{4D\tau+w_{eff}^2}}$$

where A is the ratio between the longitudinal and radial waist, D is the diffusion coefficient, r is the distance between the detectors and

$$w_{eff}^2 = \frac{w_1^2 + w_2^2}{2}$$

is the effective waist. The effective waist is computed as a combination of the waist of the two detectors, obtained by the calibration, as well as the distance between each detector. In this application,

we utilized the cross-correlation between groups of three detectors, rather than single detectors, in order to increase the signal to noise ratio.

With this information, we can fit the diffusion coefficient, which represents the diffusion coefficient the probe would have when diffusing with Brownian motion between the two detectors. This value can be slower if the probe encounters obstacles to the motion located along the line connecting the two detectors. Since every detector returns several values of the diffusion coefficient at different angles, it is also possible to fit these results to an ellipse, using the fitted eccentricity as a measure of local anisotropy, as shown in *Supplementary Figure 4 b, d.*

Supplementary Figure 4 shows example of connectivity analysis for isotropic (*Supplementary Figure 4 a, b*) and anisotropic (*Supplementary Figure 4 c, d*) samples.

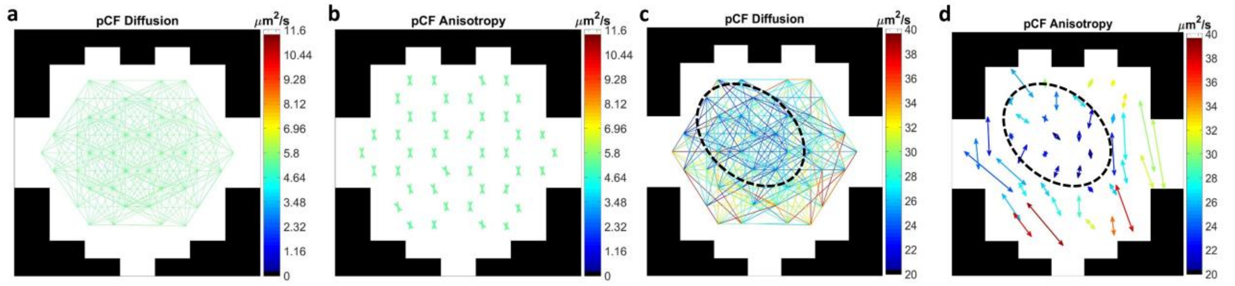

Supplementary Figure 4 Connectivity (a) and anisotropy (b) map of a sample of 20 nm diffusing beads in solution. Connectivity (c) and anisotropy (d) map of a sample of EGFP diffusing in the nucleus of NIH-3T3 cells. The segments connecting each detector and the anisotropy arrows are color-coded according to the fitted diffusion coefficient and the length of the arrow is proportional to the eccentricity in that specific point. The region highlighted in c and d show the presence of an obstacle which results in a lower connectivity (c) and an isotropic diffusion inside the obstacle (d), while showing a faster and directed flux of molecules around it (c-d).

Criteria of cell regions selection

NIH-3T3 cells have a distinctive pattern of chromatin organization, showing a segregation of the heterochromatin in spot-like regions in the nucles[3,4] that can be easily detected with a DNA staining. In the nucleus, we call these spot-like regions heterochromatin-rich regions, while we call euchromatin-rich regions the regions in which the nuclear stain signal is low and the EGFP intensity is high. The regions in which the EGFP signal is low and the DNA stain is not present are the nucleoli, clearly outlined by a peri-nucleolar heterochromatin ring.

In the cytoplasm the diffusion of EGFP can be broadly divided into two regions that we name structured an unstructured cytoplasm. The unstructured cytoplasm is present in regions poor in cytoskeleton and organelles, typically far from the nucleus, that displays a high and homogeneous EGFP signal. We refer to structured cytoplasm the region of the cytoplasm closer to the nuclear envelope, in which the organelles are present and the cytoskeleton is typically more packed.

Note on average intensity differences

Intensity differences between single detectors are substantial, therefore is important to state how, and if, those differences affect the analysis presented in this work.

Average intensity differences will affect the fluorescence fluctuation analysis only in techniques that exploit cross-correlation functions between different detectors, namely iMSD and 2D-pCF. In our implementation iMSD is not influenced since we obtain the spatiotemporal correlation function from detectors placed at the same distance from the center of the optical path, therefore differences in shape of the PSF and the average intensity are negligible. The same is true for the 2D-pCF analysis, which is implemented in such a way that the pair correlation function computed for every distance is obtained by the cross-correlation between the central detector and a group of detectors placed at the same distance from the center of the optical path.

Finally, the connectivity map is obtained by individually fitting the cross-correlation function of each pair of detectors using the values for the single PSFs obtained from the calibration and a value of diffusion time is obtained for each pair. In this framework, differences in intensity will affect only the amplitude of the function, which is not considered in the analysis.

SUPPLEMENTARY REFERENCES

1. Dertinger, T. *et al.* The optics and performance of dual-focus fluorescence correlation spectroscopy. *Opt. Express* 16, 14353 (2008).
2. Müller, C. B., Eckert, T., Loman, A., Enderlein, J. & Richtering, W. Dual-focus fluorescence correlation spectroscopy: A robust tool for studying molecular crowding. *Soft Matter* 5, 1358–1366 (2009).
3. Nielsen, A. L. *et al.* Interaction with members of the heterochromatin protein 1 (HP1) family and histone deacetylation are differentially involved in transcriptional silencing by members of the TIF1 family. *EMBO J.* 18, 6385–6395 (1999).
4. Goodarzi, A. A., Noon, A. T. & Jeggo, P. A. The impact of heterochromatin on DSB repair. *Biochem. Soc. Trans.* 37, 569–576 (2009).
